# Counting and mapping of subwavelength nanoparticles from a single shot scattering pattern

**DOI:** 10.1515/nanoph-2022-0612

**Published:** 2023-01-18

**Authors:** Eng Aik Chan, Carolina Rendón-Barraza, Benquan Wang, Tanchao Pu, Jun-Yu Ou, Hongxin Wei, Giorgio Adamo, Bo An, Nikolay I. Zheludev

**Affiliations:** Centre for Disruptive Photonic Technologies, The Photonics Institute, School of Physical and Mathematical Sciences, Nanyang Technological University, 637371 Singapore, Singapore; Centre for Photonic Metamaterials and Optoelectronics Research Centre, University of Southampton, Southampton SO17 1BJ, UK; School of Computer Science and Engineering, Nanyang Technological University, 639798 Singapore, Singapore

**Keywords:** nanoparticle counting, nanoparticle imaging, sub-Rayleigh counting

## Abstract

Particle counting is of critical importance for nanotechnology, environmental monitoring, pharmaceutical, food and semiconductor industries. Here we introduce a super-resolution single-shot optical method for counting and mapping positions of subwavelength particles on a surface. The method is based on the deep learning analysis of the intensity profile of the coherent light scattered on the group of particles. In a proof of principle experiment, we demonstrated particle counting accuracies of more than 90%. We also demonstrate that the particle locations can be mapped on a 4 × 4 grid with a nearly perfect accuracy (16-pixel binary imaging of the particle ensemble). Both the retrieval of number of particles and their mapping is achieved with super-resolution: accuracies are similar for sets with closely located optically unresolvable particles and sets with sparsely located particles. As the method does not require fluorescent labelling of the particles, is resilient to small variations of particle sizes, can be adopted to counting various types of nanoparticulates and high rates, it can find applications in numerous particles counting tasks in nanotechnology, life sciences and beyond.

## 1 Introduction

Particle counting is of crucial importance for many industries. Production of semiconductor circuits and optical components require high purity gases and liquids for chemical etching, deposition, oxidation, doping, mask removal and polishing, critical cleaning and rinsing steps in many nanotechnology processes while contaminations impact yield and throughput. Life science applications, pharmaceutical and biotech manufacturing, medical devices, cosmetics production, and food processing necessitate strict control of particulate and microbial aerosol burden to reduce the risk of contamination to products. In applications including oil, fuels, hydraulic fluids, counting of particles is important to avoid failure of bearings, pumps and seals. Painting automobiles in clean environments reduces the need to rework defects in paint finishes.

In industrial environments, particle counting is commonly based on detecting light blocking or scattering by particles in the flow of liquid or gas [1]. For more quantitative measurements, direct counting of particles by human operators or sophisticated software from the images of particle groups taken by either optical or scanning electron microscope (SEM) is used routinely. Methods such as PALM and STED work with photoactivated particles and can resolve the particles beyond the Rayleigh limit [2, 3], however are slow and require sophisticated and complex imaging equipment. Recent advances include the use of convolutional neural network to classify isolated nanoparticles [4].

In this paper, we report an optical method for counting and localizing subwavelength particles on a 4 × 4 grid that is principally different from previously reported methods. It is based on the analysis of the intensity profile of the scattering pattern created by the particles. It is a simple, non-invasive, single-shot technique that nevertheless allows counting closely placed subwavelength particles. The technique counts particles that are smaller than the diffraction limit of resolution of optical microscopes and are spaced by less than the Rayleigh distance at which particles are not individually resolved. In contrast to the traditional methods of solving the inverse scattering problem using numerical or heuristic methods in which careful choice of regularization based on extensive domain knowledge is needed [5–8], our technique depends on the artificial intelligence analysis of light diffracted on the particles in the form of deep learning. Recently, it was shown that a well-trained neural network can be used as an efficient tool for solving the Fredholm integral equations to which the inverse scattering problem can be reduced [9, 10]. The neural network approach to the analysis of scattered light has already been used in far-field low-dimensional optical metrology with resolution exceeding one percent of optical wavelength [11] and far beyond [12]. In contrast to metrology, counting and mapping is a more complex, higher-dimension task of finding the number and positions of several particles in a group retrieved from a single-shot intensity diffraction pattern of coherent light illuminating it. The retrieval is performed by an artificial neural network trained on a large number of particle groups of *a priori* known configurations. In the proof-of principle experiments, we counted and localized subwavelength holes in a metal film.

## 2 Super-resolved counting of subwavelength nanoparticles

**Figure 1:**
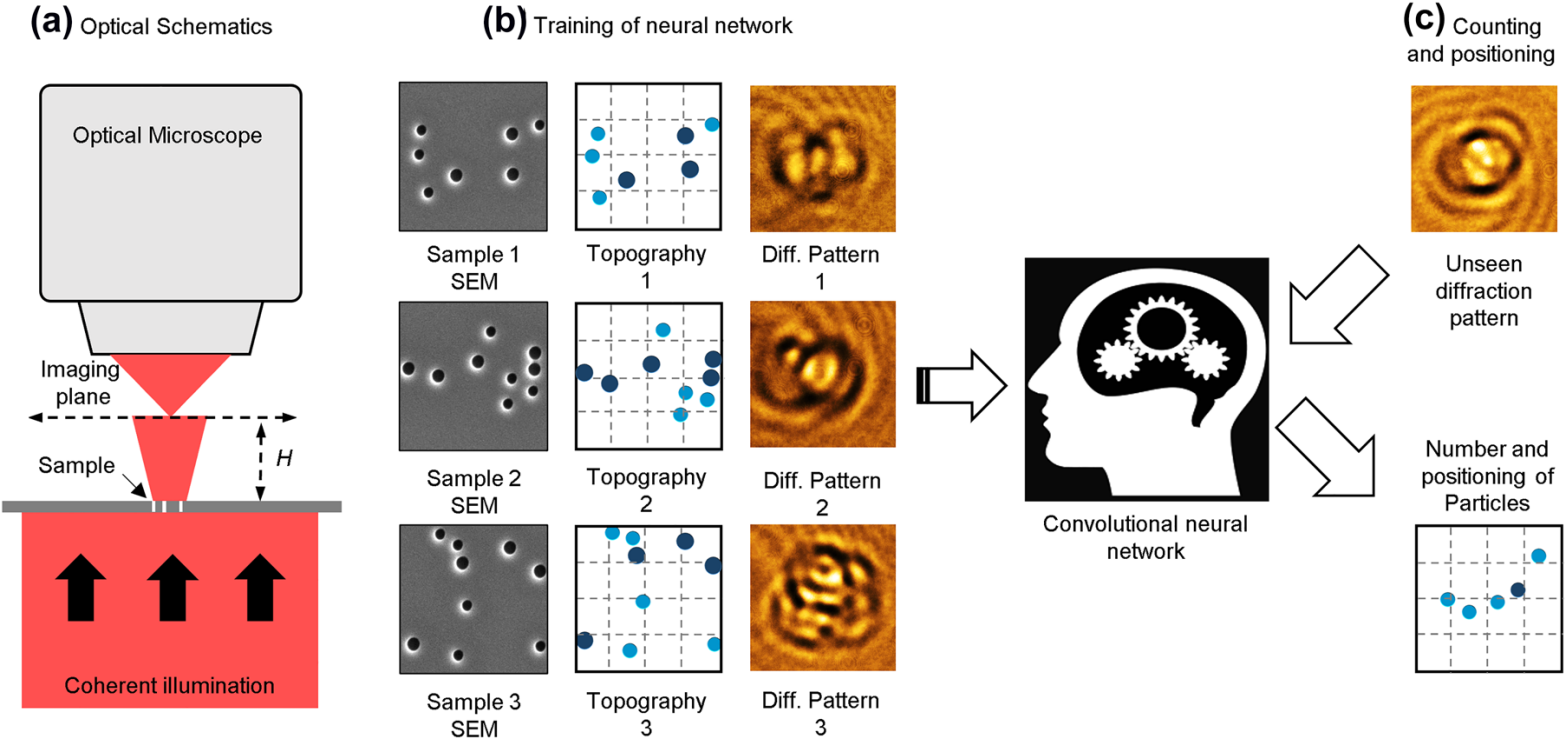
Counting and localization of subwavelength nanoparticles from their diffraction pattern. (a) Optical schematic of the technique; (b) examples of three different elements of the training set each containing a SEM image of holes in a thin chromium film on a 3.2*λ* × 3.2*λ* field of view, a corresponding topography map and a diffraction pattern are shown. The light and dark blue dots indicate the position of holes with diameters *λ*/2.6 and *λ*/3.2 correspondingly; (c) when presented with an unseen diffraction pattern, the trained convolutional neural network is able to retrieve the number of the holes in the field of view and map them on a 4 × 4 grid (indicated by the dashed lines).

The experiments described below were performed using a transmission optical microscope with a total magnification of ×300 and coherent laser illumination at the wavelength of *λ* = 633 nm (He–Ne laser). The diffraction patterns created by a group of subwavelength holes were imaged at a distance *H* = 2*λ* from the sample by a lens with numerical aperture NA = 0.9 (Figure 1a). Using focused ion beam milling we have manufactured 12,000 unique sets of holes in a 50 nm thick chromium film. Each set contained up to 10 holes randomly located in the area of 3.2*λ* × 3.2*λ* in size (field of view). To achieve closer proximity to the real-life conditions where particles could have a variation of sizes, the holes’ diameters were chosen randomly to be either *λ*/2.6 or *λ*/3.2. From the set of 12,000 different groups of holes, 70% were used for the neural network training and validation while the remaining 30% of the sets, yet unseen by the neural network were used to evaluate the accuracy of counting.

**Figure 2:**
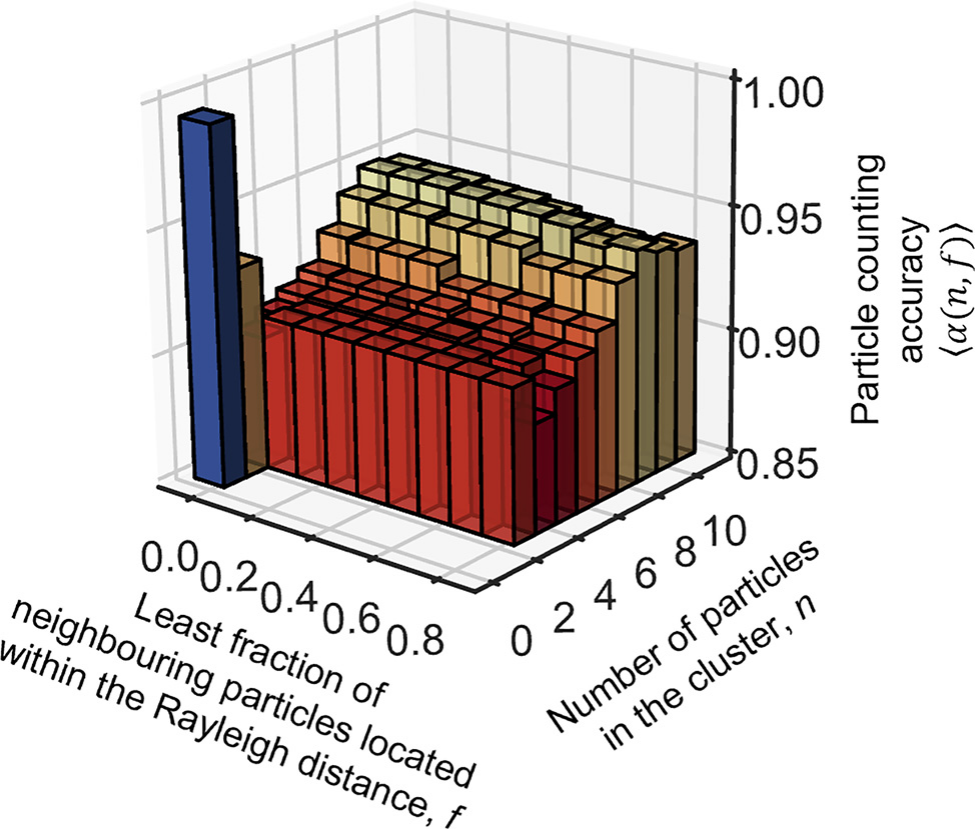
Counting subdiffraction particles. Accuracy of counting ⟨*α*(*n*, *f*)⟩ is presented as a function of number of particles *n* and least fraction *f* of neighbouring particles located within the Rayleigh distance in the group.

We define the error of particle counting as $\varepsilon =\left|n^{\prime }-n\right|/n$ , where *n*′ is the number of particles in the group counted optically and *n* is the factual number of particles in the group. The accuracy of counting for an individual counting event will then be defined as *α* = 1 − *ɛ*. Its average value ⟨*α*(*n*)⟩ represents accuracy of counting for an ensemble of particle groups containing *n* particles each. Naturally, accuracy of counting depends on the distribution of particles within the field of view. Sparsely distributed particles shall be easier to count. Particles located closer than the Rayleigh distance between their centers *r* = 0.61*λ*/NA, where NA is the numerical aperture of the imaging lens, are not resolvable by a conventional microscope. They shall be more difficult, or impossible to count from a conventional image. To judge if our technique provides super-resolution, i.e. can count closely spaced particles, it is important to know what fraction of particles in the group is located within the Rayleigh distance. To quantify the ability to count closely located particles we calculate the average accuracy of super-resolved counting ⟨*α*(*n*, *f*)⟩ as accuracy of counting in the groups where the fraction *f* of neighboring particles located within the Rayleigh distance is larger than *f*. Results on accuracy of counting derived from examining 3600 random samples are presented in Figure 2.

From Figure 2, it is evident that the accuracy of counting depends on *n*, the number of particles in the group and *f*, the proportion of particles located closely, within the Rayleigh distance. Absence of particles ⟨*α*(0, 0)⟩ = 0.99 and presence of only one particle ⟨*α*(1, 0)⟩ = 0.93 are detected with high accuracy. If the particles are all spaced more than by Rayleigh distance, i.e. *f* = 0, with an increase of the number of particles from *n* = 2 to *n* = 9, the accuracy gradually increases from ⟨*α*(2, 0)⟩ = 0.902 to ⟨*α*(9, 0)⟩ = 0.947, and falls a little to ⟨*α*(4, 0)⟩ = 0.934 for *n* = 10 particles in the group. What we found quite unexpected is that accuracy reduces insignificantly with increased fraction of particles *f* that are closely spaced particles in the group, within the Rayleigh distance. In our experiment, the holes can be as close as 0.38*λ* for a pair of smallest particles and as close as 0.31*λ* for a pair of the largest non-overlapping holes, but they are still countable with accuracy between 0.93 and 0.94. For instance, ⟨*α*(9, 0)⟩ = 0.947 falls only to ⟨*α*(9, 0.8)⟩ = 0.939 when at least 80% of particles in the group are spaced within the Rayleigh distance of *r* = 0.68*λ*. We observed that the overall intensity of the image does not significantly depend on the number of particles and therefore the network could not learn the number of particles from intensity of the images. We therefore argue that our technique allows high accuracy super-resolved counting of subwavelength particles far exceeding what is possible with a conventional microscope.

## 3 Mapping of nanoparticles on a 4 × 4 grid

Besides super-resolved counting of subwavelength particles, we have demonstrated the ability to identify these particles’ positions on a 4 × 4 grid. This is to say that we demonstrated 16-pixel binary imaging of the particle ensemble. The 3.2λ × 3.2λ field of view with a 4 × 4 grid has a pixel 0.79λ × 0.79λ in size, which for the lens used in the experiment is only 16% larger than the Rayleigh distance of 0.68*λ*. Here, from 12,000 different groups we used 70% for training and validation of the neural network and 30% to test accuracy of localization.

**Figure 3:**
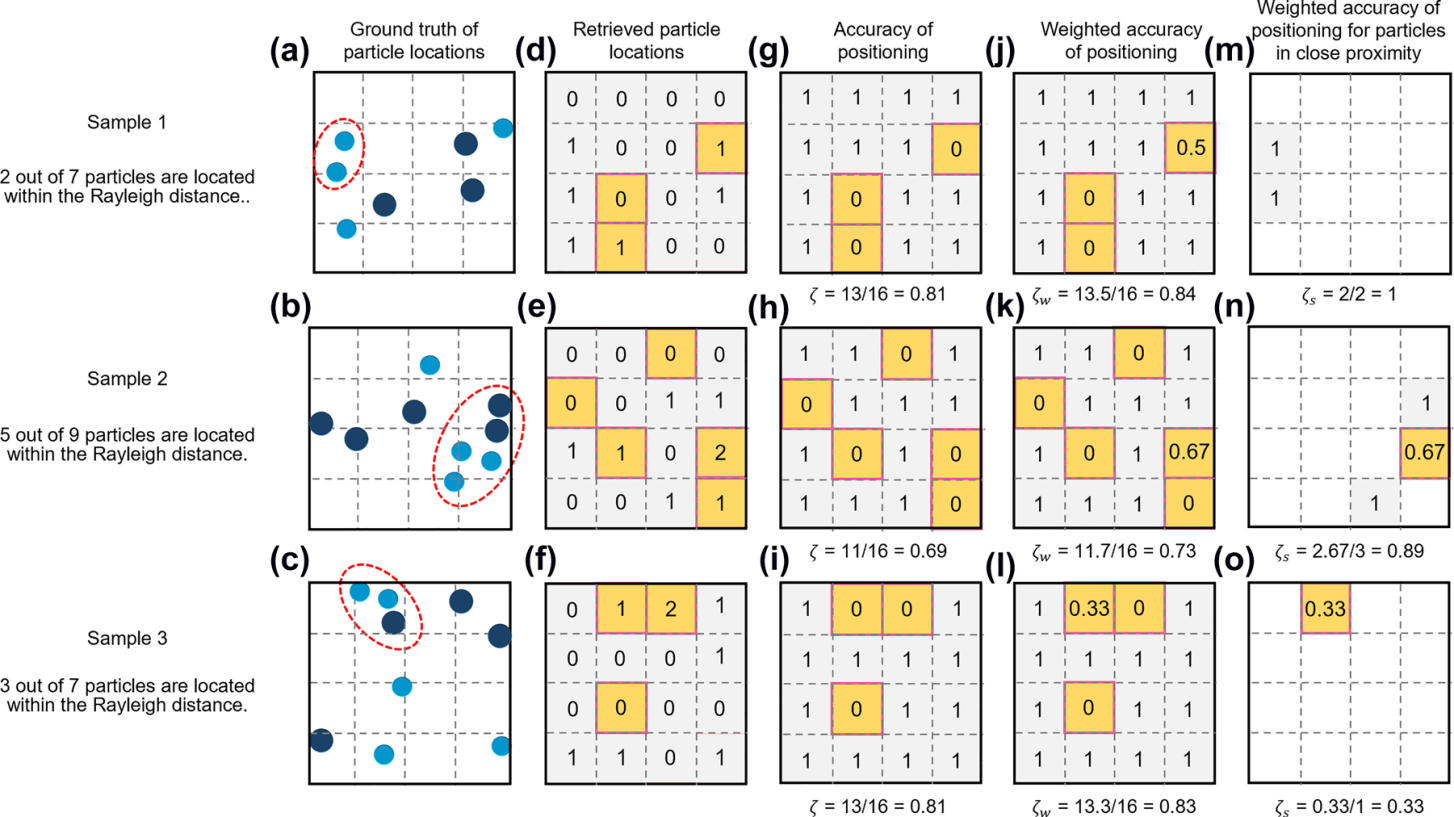
Evaluating accuracy of mapping particles on a grid. (a–c) Three typical example of particle distributions in the field of view on a 4 × 4 grid. The light and dark blue dots indicate position of holes with diameters *λ*/2.6 and *λ*/3.2 correspondingly. Particles within the red-dashed ellipses have neighbours located closer than the Rayleigh distance; (d–f) optical retrieved particle locations. Grey and yellow filling indicates pixels with correctly and incorrectly retrieved number of particles, respectively; (g–i) accuracy of identifying particle positions *ζ*; (j–l) weighted accuracy of positioning *ζ*_*w*_; (m–o) weighted accuracy of positioning *ζ*_*s*_, only for the pixels containing closely located particles.

A particle belongs to a certain pixel if its centre is within the pixel perimeter. All pixels are numbered by index *i*. To quantify accuracy of identification of the particle positions on the grid, we calculated *ζ* = *K*′/*K* where *K*′ is the number of pixels with correctly identified number of particles and *K* is the total number of pixels (*K* = 16 in the case of 4 × 4 grid). The more representative, weighted accuracy of positioning $\zeta_{w}=\frac{1}{K}\sum_{i=1}^{i=K}\rho_{i}\left(m^{\prime },m\right)$ is the ratio between the cumulative recognition weights *ρ*_*i*_(*m*′, *m*) for all pixels of the image over the total number of pixels, where *m*′ is the number of particles in the pixel counted optically and *m* is the factual number of particles in the pixel. Here *ρ*_*i*_(*m*′, *m*) is calculated accordingly to Table 1, which accounts to how well the number of particles is counted in individual pixels. To quantify the super-solution capability of the technique, we evaluated *ζ*_*s*_, the weighted accuracy of positioning calculated only for the pixels that contain particles located within the Rayleigh distance. Typical examples of retrieval of particle position in a single group are presented in Figure 3.

**Table 1:** Weight coefficient *ρ*(*m*′, *m*) is a measure, of how accurately the number of particles in each pixel is retrieved.

	*m*′, the number of particles in the pixel counted optically				
		0	1	2	3	4
*m*, the factual number of particles	0	1	0	0	0	0
	1	0	1	1/2	1/3	1/4
	2	0	1/2	1	2/3	2/4
	3	0	1/3	2/3	1	3/4
	4	0	1/4	2/4	3/4	1

**Figure 4:**
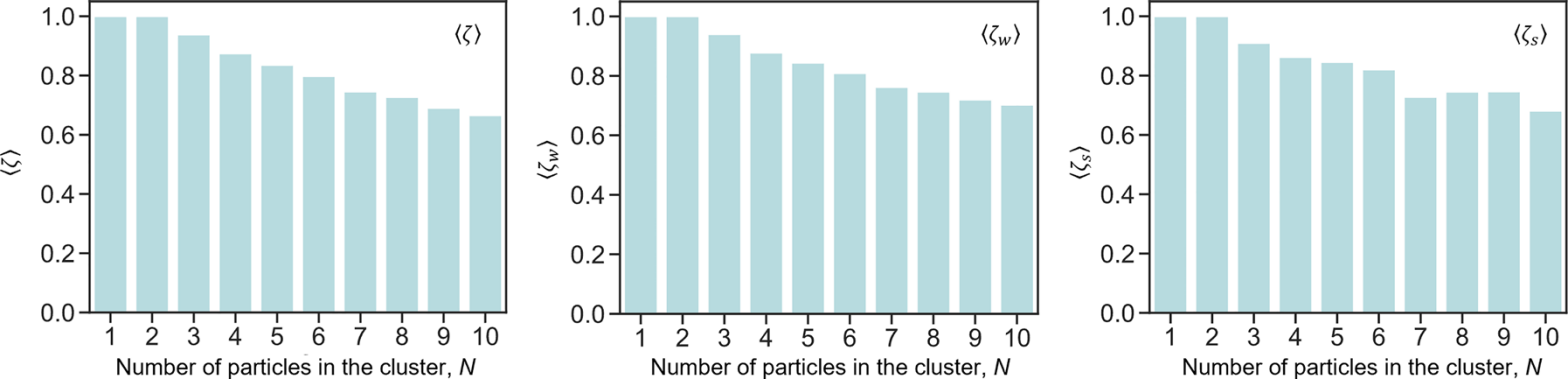
Accuracy of mapping particles for groups of different sizes. (a) Ensemble average accuracy of positioning, ⟨*ζ*⟩, (b) ensemble average weighted accuracy of positioning ⟨*ζ*_*w*_⟩; (c) ensemble average weighted accuracy of positioning calculated only for the pixels, containing particles located within the Rayleigh distance, ⟨*ζ*_*s*_⟩.

The ensemble average values of ⟨*ζ*⟩, ⟨*ζ*_*w*_⟩ and ⟨*ζ*_*s*_⟩ calculated for the ensample of 3600 particles are presented on Figure 4. In the groups containing only one or two particles, positions of the particles in the pixel grid can be identified with nearly perfect accuracy. Uncertainty over the particle positions accumulates with the group size: ⟨*ζ*⟩, ⟨*ζ*_*w*_⟩ slowly decay with increased number of particles in the group reaching the level of 0.65 for 10 particles in the group. Importantly, close proximity of the particles (located within the Rayleigh distance) does not reduce the accuracy of positioning. This is witnessed by a comparison of ⟨*ζ*_*s*_⟩ and ⟨*ζ*⟩, ⟨*ζ*_*w*_⟩ curves, confirming that mapping of particle positions is achieved with super-resolution.

## 4 Conclusions

In this work, we have introduced a non-invasive single-shot super-resolved optical method for counting and mapping positions of subwavelength particles on a 4 × 4 grid from their scattering patterns. Although the method has been demonstrated in a proof-of-principle experiment with “negative” particles, subwavelength holes in opaque screen, we argue that it is suitable for “positive” particles in particular with polarization contrast or total internal reflection microscopy modes suppressing the background light. In practical applications of the technique, for particles of known shape, training of the network for the deep learning process used in counting and localization of the particles can be achieved with computer-generated sets comprising of a large number of random groups and corresponding computed scattering patterns. We therefore argue that the technique can be used on a large class of nanoparticles of known shape that are not countable from their optical images because of their small sizes and close proximity. Moreover, the single-shot nature of the techniques shall allow a high-speed counting which is limited only by the frame rate of image sensor, which currently reaches hundreds of millions of frames per second.

## Appendix A

### A.1 Artificial neural network

In the Super-resolved counting of subwavelength nanoparticles section of the main text, the diffraction images of the nanoholes normalized to an average mean of 0 and standard deviation of 1 and nanoholes counts labels are used to train a 34 convolutional Residual network. The neural network is trained using Pytorch libraries. Default Residual network design rules: filter kernel size of 3 × 3, shortcut connection every bi-adjacent layers, batch-normalization after each convolutional layer, followed by activation function, and downsampling directly by a convolutional layer with a stride of 2 after every 6 convolutional layers are implemented. The network ends with a global average pooling layer, a 512 neurons fully connected layer and 1 nanoholes counts output. ReLU activation is used in the convolutional blocks. After the 1 neuron output, we include a non-trainable integer rounding layer to obtain the nanoholes counts. The weights of the network is pretrained with ImageNet database. A mini-batch size of 128 is used and we used Adam optimization with default initialization and decoupled weight decay regularization. The learning rate is adjusted with a pre-defined step decay schedule by a factor of 0.8. We use mean absolute error between the correct nanoholes count and measured nanoholes count as the regression loss function. The network is trained for 500 epochs. Network at epochs with the lowest mean absolute error in the validation set is used to measure nanoholes in the test set.

In the mapping of nanoparticles on a 4 × 4 grid of the main text, we adapt the artificial neural network output to retrieve the number of nanoholes in a 4 × 4 array. The convolutional Residual blocks are kept the same while we connect a 2 layer deep fully connected layers [512–128 neurons] followed by the 4 × 4 neurons output. ReLU activation is used in the fully connected layers and Linear activation is used before the output. Similar with the counting task, we include a non-trainable layer to obtain the nanoholes counts in each grid. The training initialization and optimization settings are kept the same.
